# Influences of cold atmospheric plasma on apoptosis related molecules in osteoblast-like cells *in vitro*

**DOI:** 10.1186/s13005-021-00287-x

**Published:** 2021-09-03

**Authors:** Benedikt Eggers, Jana Marciniak, Svenja Memmert, Gunar Wagner, James Deschner, Franz-Josef Kramer, Marjan Nokhbehsaim

**Affiliations:** 1 Department of Oral, Maxillofacial and Plastic Surgery, Center of Dento-Maxillo-Facial Medicine, University Hospital Bonn, Welschnonnenstr. 17, 53111 Bonn, Germany; 2 Section of Experimental Dento-Maxillo-Facial Medicine, Center of Dento-Maxillo-Facial Medicine, University Hospital Bonn, Welschnonnenstr. 17, 53111 Bonn, Germany; 3 Department of Orthodontics, Center of Dento-Maxillo-Facial Medicine, University Hospital Bonn, Welschnonnenstr. 17, 53111 Bonn, Germany; 4 Department of Periodontology, Operative and Preventive Dentistry, Center of Dento-Maxillo-Facial Medicine, University Hospital Bonn, Welschnonnenstr. 17, 53111 Bonn, Germany; 5 Department of Periodontology and Operative Dentistry, University Medical Center of the Johannes Gutenberg University, Augustusplatz 2, 55131 Mainz, Germany

**Keywords:** Cold atmospheric plasma, Apoptosis, Inflammation, Bone remodelling, Osteoblast like cells

## Abstract

**Background:**

Cold atmospheric plasma (CAP) has recently been identified as a novel therapeutic strategy for supporting processes of wound healing. Since CAP is additionally known to kill malignant cells, our study intends to determine the influence of CAP on crucial molecules involved in the molecular mechanism of apoptosis in osteoblast-like cells.

**Methods:**

Human osteoblast-like cells were CAP-treated for 30 and 60 s. CAP effects on critical factors related to apoptosis were studied at transcriptional and protein level using real time-PCR, immunofluorescence staining and western blot. Phalloidin / DAPI staining was used for analyzing the cell morphology. In addition, apoptotic outcomes of CAP were displayed using flow cytometry analysis. For studying intracellular signaling pathways, MAP kinase MEK 1/2 and PI3K were blocked. Finally, the effects of CAP on caspase-3 activity were examined using a caspase-3 assay.

**Results:**

CAP treatment resulted in a significant downregulation of p53 and apoptotic protease activating factor (APAF)-1, caspase (CASP)9, CASP3, BCL2 Antagonist/Killer (BAK)1, and B-Cell Lymphoma (BCL)2 mRNA expression at 1 d. An inhibitory effect of CAP on apoptotic genes was also shown under inflammatory and apoptotic conditions. Nuclear translocation of p53 was determined in CAP treated cells at the early and late stage, after 15 min, 30 min, and 1 h. p53 and APAF-1 protein levels were reduced at 1 d, visualized by immunofluorescence and western blot, respectively. Moreover, a morphological cytoskeleton modification was observed after CAP treatment at 1 d. Further, both CAP-treated and untreated (control) cells remained equally vital as detected by flow cytometry analysis. Interestingly, CAP-associated downregulation of CASP9 and CASP3 mRNA gene expression was also visible after blocking MAP kinase and PI3K. Finally, CAP led to a decrease in CASP3 activity in osteoblast-like cells under normal and apoptotic conditions.

**Conclusions:**

Our in vitro-study demonstrated, that CAP decreases apoptosis related molecules in osteoblast-like cells, underlining a beneficial effect on hard-tissue cells.

## Background

The healing of hard and soft tissue areas is a complex interplay between many upregulated factors in order to regenerate the former anatomic structure. Especially, the healing of bone is a complicated and long-term process, involving many different mechanisms like the growth of new blood vessels, the aggregation of mesenchymal stem cells, the differentiation of osteoblasts and the formation of the extracellular matrix [1, 2]. Whenever the wound healing does not take place in a normal way, which can occur at multiple steps along the cascade, a chronic wound may result. Apart from the fact of being an enormous psychological burden for the patient, the continuous treatment is also expensive for the healthcare system [3]. Chronic wounds have a high proportion of inflammatory, damaged or dead cells, which are usually cleared by macrophages [4]. Another important process involved in damaged cells removal is apoptosis also known as programmed cell death. Apoptosis can be induced by an extrinsic and intrinsic pathway [5, 6]. The extrinsic pathway can be initiated by molecules such as Tumor Necrosis Factor (TNF)α, which modulate an apoptotic cascade, which finally leads to the activation of CASP3. The intrinsic pathway can be triggered by cell stress, which leads to the activation of reactive oxygen species (ROS) or the release of p53 caused by DNA damage. The process results in the release of apoptogenic factors, such as cytochrome c, and apoptotic protease activating factor (APAF)-1, and an activation of caspase (CASP)9 and CASP3. CASP3 activation causes instability of cell membranes and fragmentation of DNA [7–9]. The apoptotic cascade is further modulated by anti-apoptotic genes, such as BCL2 Antagonist/Killer (BAK)1, or anti-apoptotic genes, such as B-Cell Lymphoma (BCL)2 [10].

Currently, CAP, being generated by inert gas or the ambient air as the fourth state of matter, has been shown to improve wound tissue healing - it seems to be a promising therapeutic approach for non-healing inflammatory wounds [11–13]. Though the specific effects of CAP need to be unrevealed, the CAP treatment of tissues seems to trigger a number of cellular mechanisms: *in vitro*-studies show a stimulating effect of CAP on human periodontal cells, keratinocytes and fibroblasts by upregulating certain genes and increasing cell migration and viability [14–17]. Additionally, CAP has been shown to enhance cell adhesion onto pre-treated surfaces [18–21]. However, apoptotic effects of CAP have also been described in the literature, which might represent promising approaches in cancer-therapy [22, 23]. CAP promotes the development of ROS and reactive nitrogen species which can induce apoptosis in some types of malignant cells via the intrinsic pathway as described above [24, 25]. Interestingly, CAP does not seem to affect healthy cells [26].

In our recent study we demonstrated, that CAP positively influences the wound healing in human osteoblast-like cells by stimulating proliferation and by increasing cell migration and viability [27]. However, the underlying molecular mechanisms of CAP on cell death in healthy cells are still unknown. Therefore, the aim of the present study was to analyze the effect of CAP on cell viability and death in this primary human osteoblast cell line, in order to study the effects of CAP-treatment on hard tissue.

## Methods

### Cell culture and treatment

Human osteoblast-like cells (ATCC, CRL-1427^™^; Sigma-Aldrich, Taufkirchen, Germany) were cultured in Dulbecco’s modified essential medium (DMEM, Invitrogen, Germany) supplemented with 10 % fetal bovine serum (FBS, Invitrogen), 100 units penicillin, and 100 µg/mL streptomycin (Invitrogen) at 37^°^C in a humidified atmosphere of 5 % CO_2_ and 95 % humidity. For further studies, cells were seeded into 35 × 10 mm petri dishes and cultured to 70 % confluence. Cell culture medium was replaced every 2 days. For each experiment, FBS concentration was reduced to 1 % one day prior to the start of the experiment.

### CAP application

CAP was generated by a dielectric barrier discharge (Plasma ONE MEDICAL, Plasma MEDICAL SYSTEMS® GmbH, Nievern, Germany). Pulsed direct current (35 V) is transformed to high voltage resulting in an electric field, which forms the plasma. The CAP can be used at five levels of intensity (20 %, 40 %, 60 %, 80 %, 100 %), modulating the high voltage (3–18 kV). Osteoblast-like cells were exposed to CAP for 60 s as previously described [17]. Optimal time, intensity and distance were selected after preliminary experiments. To analyze the CAP effects in an inflammatory or apoptotic environment *in vitro*, in a separate experimental set, cells were pre-treated prior to the CAP application either with human recombinant interleukin (IL)-1β (PromoKine, Heidelberg, Germany; 1 ng/ml) or with staurosporine (STS, Sigma-Aldrich; 10 nM) one h before CAP application.

### Analysis of gene expression

24 h after CAP application total RNA was extracted using an RNA extraction kit (Qiagen, Hilden, Germany). RNA (1 µg) was reverse transcribed into cDNA by use of iScript™ Select cDNA Synthesis Kit (Bio-Rad Laboratories, Munich, Germany) at 42^°^C for 90 min followed by 85^°^C for five min. One µl of cDNA was amplified as a template in a 25 µl reaction mixture containing 12.5 µl SsoAdvanced™ Universal SYBR^®^ Green Supermix (Bio-Rad), 2.5 µl of specific commercially available primers (0.5 µM each; predesigned QuantiTect Primer Assay, Qiagen), and 9 µl deionized water. The mixture was at first heated at 95^°^C for 5 min, and then followed by 40 cycles with denaturation at 95^°^C for 10 s and combined annealing/extension at 60^°^C for 30 s. Expressions of p53, APAF-1, CASP9, CASP3, BAK1 and BCL2 mRNA were detected by real-time PCR using the iCycler iQ™5 detection system (Bio-Rad). Glyceraldehyde 3-phosphate dehydrogenase (GAPDH) was used as an endogenous control. The data were analyzed by the comparative threshold cycle method.

### Analysis of p53 nuclear translocation

Osteoblast-like cells were seeded on glass coverslips (Thermo Fisher Scientific Inc., Schwerte, Germany) and propagated until 70 % of confluence was achieved. Cells were treated with CAP as described above. After 15 min, 30 and 60 min cells were washed twice with 1x PBS (Invitrogen) and fixed with 4 % paraformaldehyde (Sigma-Aldrich) at pH 7.4 and room temperature for 10 min. After another washing step cells were permeabilized in 0.1 % Triton X-100 (Sigma-Aldrich) for 5 min. Next, the cells were washed again and blocked with 5 % BSA in PBS for 60 min to reduce the background staining. Afterwards, the cells were incubated with rabbit anti-p53 primary antibody (Abcam, Berlin, Germany; 1:200) at 4 °C overnight. Following another rinsing step, the cells were incubated with CY3-conjugated goat anti-rabbit IgG secondary antibody (Abcam; 1:1000) at room temperature for 45 min. Finally, location of p53 within the stained cells was analysed with the ZOE^™^ Fluorescent Cell Imager (Bio-Rad). The images were captured with an integrated digital 5MP CMOS camera.

### Immunoblotting assay

To study the regulatory effects of CAP on apoptotic activating factor in osteoblast-like cells, immunoblotting was performed. Cells were treated with CAP for 1 d. Whole protein lysate was prepared on ice by adding cell lysis buffer (Bio-Techne GmbH, Wiesbaden, Germany) containing freshly mixed protease inhibitor cocktail (Sigma-Aldrich) to the cells. Total protein concentration was quantified by using a BCA Protein Assay Reagent Kit (Pierce Biotechnology, Rockford, IL, USA) and a microplate spectrophotometer (PowerWave x, BioTek Instruments, Winooski, VT, USA) at 562 nm. 10 % SDS-PAGE gel was used to load an equal amount of proteins into each slot using an electrophoresis system (SE260 Mighty Small II Deluxe Mini Vertical Protein Electrophoresis Unit, Hoefer, Inc., Holliston, USA). After electrophoresis, the samples were transferred to nitrocellulose membranes (Bio-Rad Laboratories) using TE22 Mighty Small Transfer Tank (Hoefer, Inc.). Prior to primary antibody incubation, nonspecific background was blocked with 2.5 % BSA in 0.1 % TBS-T for one hour at room temperature. Subsequently, membranes were incubated with primary rabbit polyclonal anti-APAF-1 (ab2000, Abcam1:500) and anti-β-actin (ab227387, Abcam; 1:10,000) over-night at 4 °C. After several washing steps, membranes were incubated with goat anti-rabbit IgG (ab205718, Abcam; 1:10,000), an HRP-conjugated secondary antibody, for 45 min at room temperature. Detection of the immune-reactive bands was performed with the enhanced chemiluminescence (ECL) Substrate (Pierce). ImageJ software was used to quantify relative protein amounts and to normalize to β-actin levels.

### Analysis of cell morphology

Osteoblast-like cells were seeded on glass coverslips as described above. As soon as the monolayer reached 70 % of confluence, medium was replaced by reduced serum-containing medium as described above and were treated with CAP. Morphological modification of CAP-treated cells was analyzed with a double Phalloidin and DAPI staining after 24 h. Fixed and permeabilized cell monolayers were incubated with fluorescent conjugates of Phalloidin (Sigma-Aldrich, 100 µM) for 60 min in order to label the actin filaments. Next, Phalloidin working solution was aspirated, and DAPI working solution (Sigma-Aldrich, 1 µg/ml) was added for 5 min to the cells to label DNA. Finally, after another rinsing, stained cells were visualized with the ZOE^™^ Fluorescent Cell Imager (Bio-Rad) as described above. Analysis of cell morphology was performed using ImageJ software by measuring cell area and aspect ratio (major axis/minor axis). For the measurements, 5 slides each were randomly selected.

### Vybrant apoptosis assay

Cells were treated with Alexa Fluor^®^ 488 annexin V/ propidium iodide (Vybrant^®^ Apoptosis Assay Kit #2; Invitrogen) according to the manufacturer’s instructions 24 h after CAP application in order to determine the cytotoxicity of CAP by flow cytometric analysis with the Cytometer FC500 (Beckman coulter, Brea, CA, USA). STS (Sigma-Aldrich; 10 nM) treated cells served as positive control. Additionally, in a separate experimental set, controls and CAP-treated cells were also stained with the above kit components and subsequently the immunofluorescence was analyzed with the ZOE™ Fluorescent Cell Imager (Bio-Rad) at 1 h, 4 and 24 h after treatment.

### Inhibition of specific signaling pathway

To reveal the intracellular signaling pathways involved in the effects of CAP on apoptosis regulation, cells were pre-treated with different inhibitors MEK1/2 (U0126; 10 µM; Calbiochem, San Diego, CA, USA) or PI3K (LY294002; 50 µM; Sigma-Aldrich), respectively, 60 min prior to CAP exposure in an additional experimental set. Subsequently, gene regulation of CASP9 and CASP3 was analyzed by real-time PCR.

### Caspase-3 assay

Cells were cultured as previously described and treated with CAP for 30 and 60 s, respectively. Additionally, cells were treated with STS (Sigma-Aldrich; 10 nM) and after 60 min with CAP. Caspase-3 activity was quantified using the colorimetric kit Caspase-3 Assay Kit (Abcam) according to the manufacturer’s protocol at one day. A microplate spectrophotometer (BioTek Instruments) was used at 405 nm to measure the optical density.

### Statistical analysis

All experiments were performed in triplicates and repeated at least twice by calculating mean values and standard errors of the mean. GraphPad Prism Software (GraphPad Software, San Diego, USA) was used for statistical analysis with Kruskal-Wallis, and Mann-Whitney U-tests with Bonferroni-Holm correction (*p* < 0.05).

## Results

### Effect of CAP on gene expression of markers of apoptosis

**Fig. 1:**
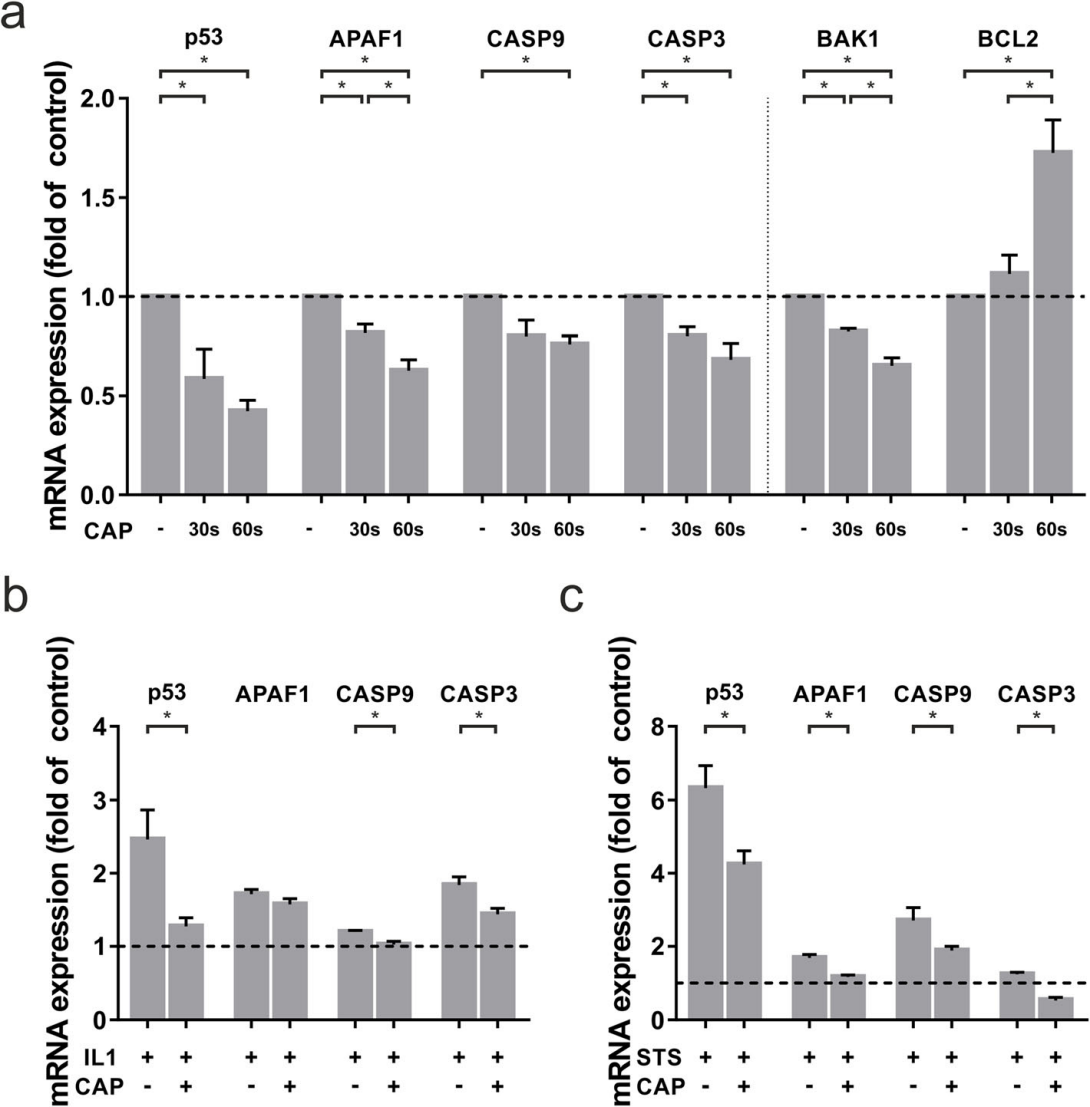
mRNA-expression of apoptotic markers in human osteoblast-like cells after 30 and 60 s of CAP treatment at 1 d as compared to untreated cells (-). **a** mRNA-expression of p53, APAF-1, CASP9, CASP3, BAK1, and BCL2, (*n* = 9). **b** mRNA-expression of p53, APAF1, CASP9, and CASP3 in IL-1β preincubated human osteoblast-like cells, (*n* = 9). **c** mRNA-expression of p53, APAF1, CASP9, and CASP3 in STS preincubated human osteoblast-like cells, (*n* = 9). * statistical significance (*p* < 0.05)

Since CAP has been described to have a stimulating effect on multiple cells, we examined a possible anti-apoptotic effect of CAP on the gene regulation in osteoblast-like cells. Regulation of critical apoptotic molecules like APAF-1, CASP9 and CASP3, and cellular tumor antigen p53, was measured at the transcriptional level 1 d after CAP application. CAP treatment resulted in a significant time-dependent decrease of p53. The effect was 20 % for 30 s and 30 % for 60 s as compared to control at 1 d. Furthermore, CAP downregulated significantly APAF-1 by 18,4 % (30 s treatment) and by 62,6 % (60 s treatment) as compared to untreated cells at 1 d. Moreover, CAP resulted in a significant downregulation of CASP9 and CASP3 by 24 % and 31,8 %, respectively, after 60 s of treatment at 1 d. Similarly, for these genes, treatment for 30 s resulted in weaker downregulation of mRNA expression level compared to 60 s. In addition, we observed CAP effects on the proapoptotic marker BAK1, for which a downregulation of gene expression by 34,8 % was observed after 60 s of treatment. Interestingly, 60 s of CAP treatment led to an upregulation of BCL2, which plays an important role in antiapoptotic processes (Fig. 1a).

### Effect of CAP on the gene expression of apoptotic molecules under inflammatory and apoptotic conditions

In order to study the effects of CAP on the gene expression on apoptotic markers in inflammation, IL-1β pre-incubated cells were treated with CAP. Our data showed, that CAP counteracted the IL-1β-induced overexpression of inflammation- and apoptosis-related genes. Under inflammatory condition, 60 s of CAP application led to a significant reduction of p53, CASP9, and CASP3 at one day. The downregulating effect was strongest for p53, which was 33 %. Another strong effect was shown for CASP3 mRNA expression: CAP treatment of IL-1β pre-incubated cells resulted in a downregulation by 21,8 %. (Fig. 1b).

Similar results were observed for STS preincubated cells. Especially in such an apoptotic environment, the significant anti-apoptotic effect of 60 s CAP treatment could be seen at the transcriptional level. The CAP induced down-regulation of p53 mRNA expression was 32,9 %. In addition, a downregulation of CASP9 and CASP3 mRNA by 30,2 % and 56,9 %, respectively, was observed after 60 s of CAP treatment at one day (Fig. 1c).

### Effect of CAP on p53 nuclear translocation and APAF-1 protein levels

**Fig. 2:**
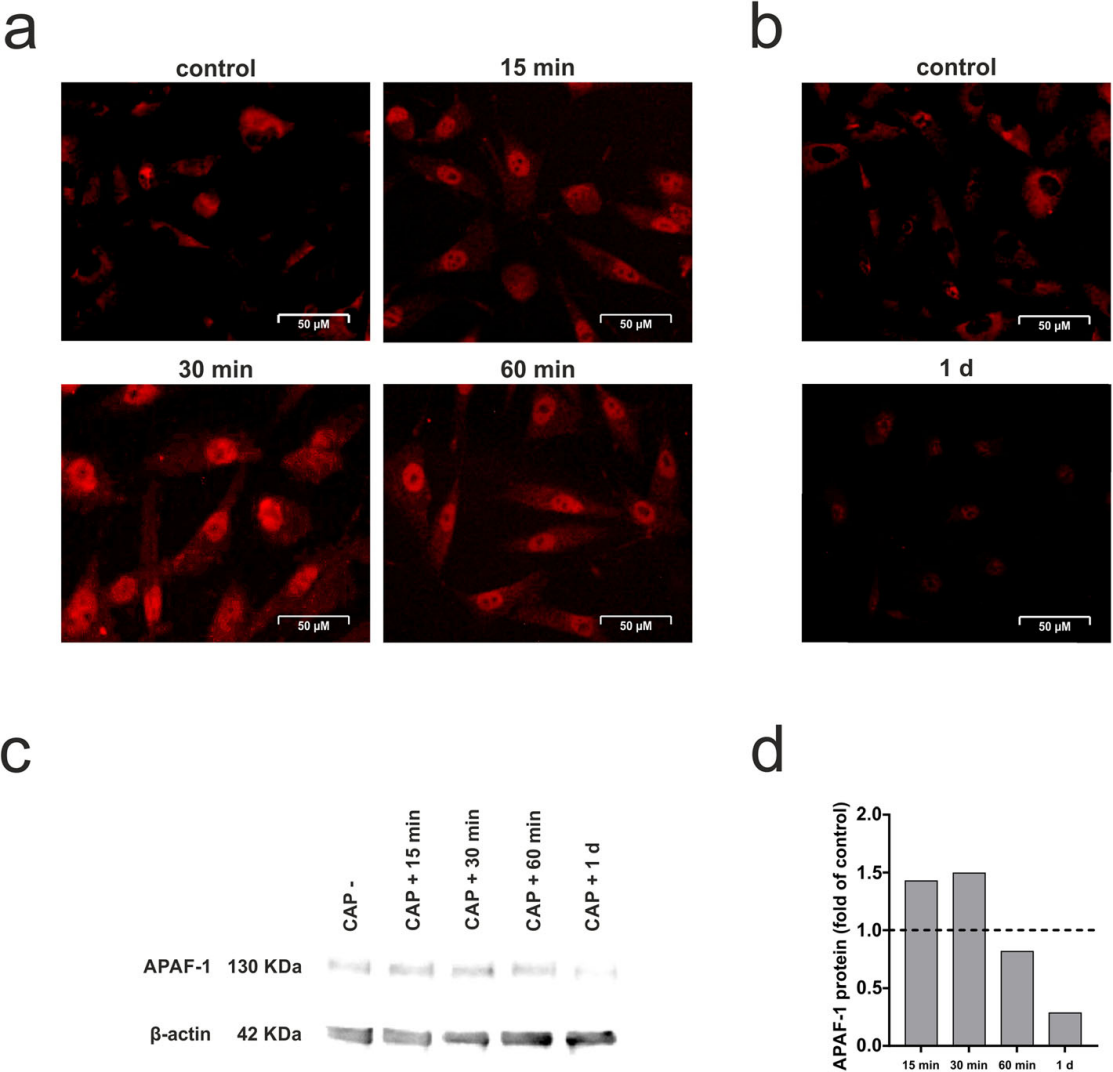
Stimulatory effect of CAP on p53 and APAF-1 levels in human osteoblast-like cells. **a** Nuclear translocation of p53 after 60 s of CAP treatment at 15 min, 30 min, 60 min as compared to untreated cells (control), (*n* = 3). **b** Immunofluorescence staining of p53 after 60 s of CAP treatment at 1 d as compared to untreated cells (control), (*n* = 3). **c** Analysis of APAF-1 protein level in total cell lysate after CAP treatment at 15 min, 30 min, 60 min and 1 d using western blot as compared to untreated cells (CAP -). Protein density values were calculated by measurement of the mean gray values for each blot, subsequent inversion and normalization to β-actin, (*n* = 3). A representative image of one experiment is shown. **d** Results of the semi-quantitative analysis performed by ImageJ software. Data were expressed as a band intensity relative to control

Additionally, a p53 nuclear translocation and accumulation was observed after CAP treatment of the cells, with the highest level at 30 min post treatment (Fig. 2a). Furthermore, p53 expression was shown by immunofluorescence at 1 d, visualizing a nearly reduced expression (Fig. 2b). By visualizing the total protein level of APAF-1 protein after CAP application, a slight increase with a peak after 30 min and a subsequent decrease could be observed (Fig. 2c).

### Influence of CAP on cell morphology

**Fig. 3:**
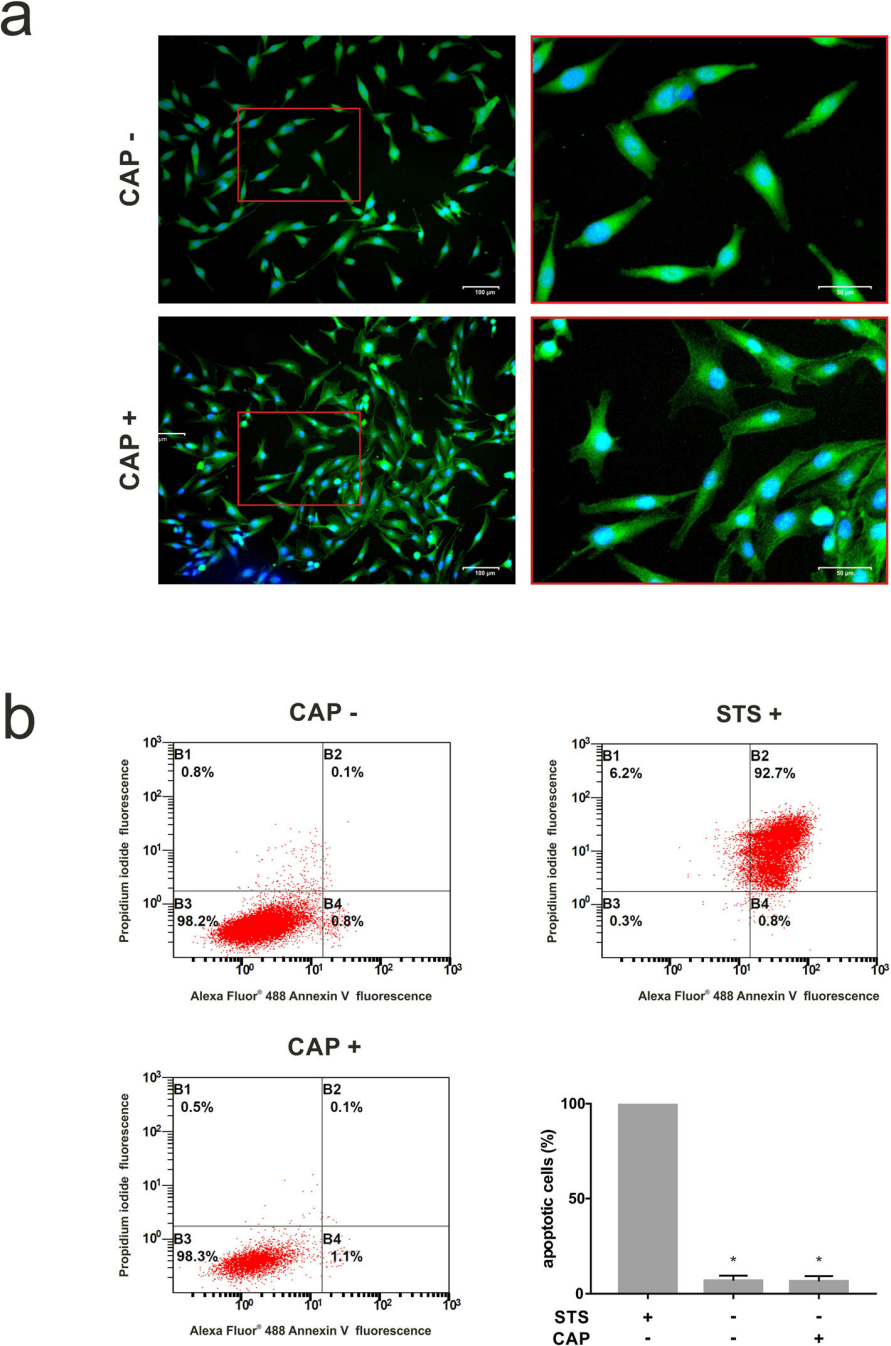
Effects of CAP application on the morphology and apoptosis of cultured human osteoblast-like cells at 1 d. **a** Actin cytoskeleton of osteoblast-like cells, untreated (CAP -) and treated (CAP +) for 60 s. Cytoskeleton and nucleus are stained with FITC conjugated phalloidin (green) and DAPI (blue), respectively, (*n* = 3). **b** Apoptotic effects in human osteoblast-like cells after 60 s of CAP treatment displayed by a Vybrant apoptosis assay. Results of flow cytometry analysis of osteoblast-like cell apoptosis after 24 h of treatment with CAP (CAP +) as compared to untreated cells (CAP -). STS preincubated cells (STS +) served as positive control. Flow cytometry was performed after cells were stained with Annexin-FITC and propidium iodide using Vybrant Apoptosis assay kit, (*n* = 3). * statistical significance to positive control (*p* < 0.05)

As visualized by fluorescence microscopy, exposure of cells to CAP caused changes of the cytoskeleton at one day (Fig. 3a). The cells changed their morphology from spindle-shaped to polygonal and had more lamellipodia and filopodia. The area of the cells increased by 38 % on average, why the aspect ratio (major axis/minor axis) decreased by 32,2 %.

### Actions of CAP on apoptosis

Furthermore, anti-apoptotic CAP influence was displayed by flow cytometry: approximately 93.5 % of cells were viable, which was similar to untreated cells (93.1 %; Fig. 3b). These results were also confirmed by immunofluorescence, demonstrating, that CAP had no regulatory effect on the numbers of apoptotic or dead cells at 1 h, 4 and 24 h after treatment (data not shown).

### Influence of CAP on specific signaling pathways

**Fig. 4:**
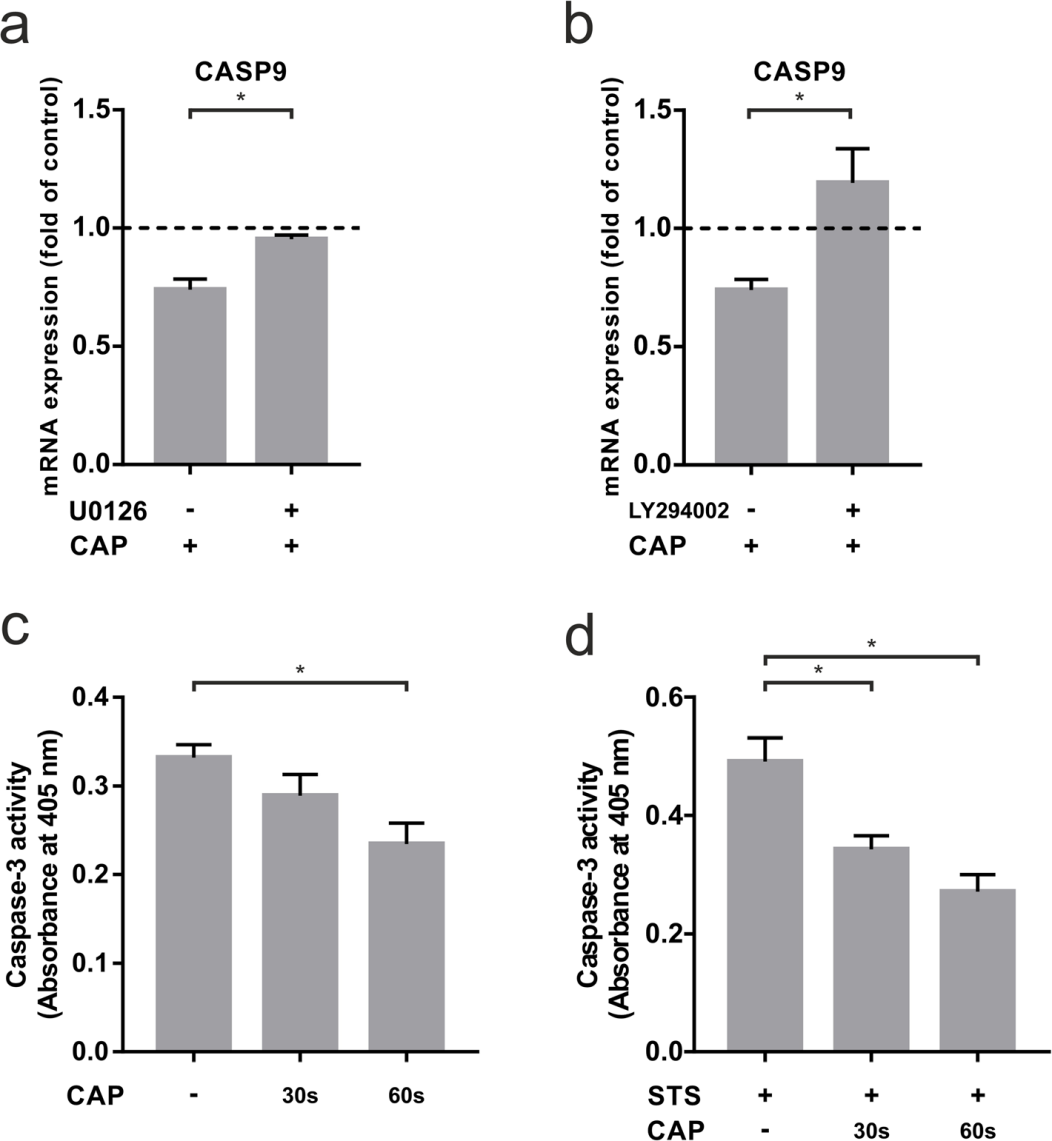
Analysis of CASP9 and CASP3 in human osteoblast-like cells after 60 s of CAP treatment at 1 d (+) as compared to untreated cells (-). **a** mRNA expression of CASP9 after preincubation with MEK 1/2 inhibitor U0126, (*n* = 9). **b** mRNA expression of CASP9 after preincubation with PI3K inhibitor LY294002, (*n* = 9). **c** Caspase-3 activity after 30 and 60 s of CAP treatment at 1 d, (*n* = 9). **d** Caspase-3 activity in STS preincubated human osteoblast-like cells after 30 and 60 s of CAP treatment at 1 d, (*n* = 9). * statistical significance (*p* < 0.05)

To better understand the intracellular mechanisms of CAP-dependent down-regulation of CASP9 and CASP3 gene expression, we incubated the cells with a MEK 1/2 and a PI3K inhibitor, both regulating anti-apoptotic processes. Blocking the MEK 1/2 pathway counteracted CAP-induced downregulation of CASP9 (Fig. 4a). Furthermore, the anti-apoptotic effect of CAP on CASP9 was also blocked after application of a PI3K inhibitor (Fig. 4b). Similar effects were observed for the gene expression of CASP3, but to a lower extent (data not shown).

### Regulation of caspase-3 activity by CAP

To confirm the above gene expression results, caspase-3 activity of CAP-treated and non-CAP-treated cells was determined. Compared to untreated cells, there was a slight time-dependent decrease in Caspase 3 activity at 1 d, which was approximately 30 % lower after 60 s of treatment (Fig. 4c). An even more significant downregulation of caspase-3 activity could be achieved by pre-incubating the cells with STS. In this apoptotic environment, caspase-3 activity was downregulated by 20 % after 30 s of CAP treatment and by 36 % after 60 s of CAP treatment at one day (Fig. 4d).

## Discussion

In the present study, CAP had been shown to down-regulate key apoptosis-related markers in osteoblast-like cells, suggesting a positive effect of CAP on hard tissue healing. Additionally, an anti-apoptotic effect of CAP in an inflammatory and apoptotic environment was shown *in vitro*. Furthermore, it has been demonstrated by DAPI/ Phalloidin staining, that CAP exposition promotes a change in cell morphology. These results imply that CAP might be a promising treatment to help to enhance wound healing of chronical hard tissue wounds.

First, we aimed to analyse the effects of CAP on gene expression of apoptotic markers in osteoblast-like cells. Therefore, we studied the expressions of the key apoptotic markers p53, APAF-1, CASP9, and CASP3 that were downregulated in CAP treated cells as compared to untreated cells. The tumour suppressor p53 is a main regulator of apoptosis induction in damaged and senescent cells and plays an important role in cellular stress responses [28]. Interestingly, the nuclear translocation of p53 was increased at 30 min after CAP application as an early cellular response to the CAP treatment. p53 activation in osteoblast-like cells seems to be enhanced as an early response after CAP treatment. However, after 24 h p53 gene expression was significantly reduced. Similar effects of CAP were observed in HaCaT keratinocytes: 15 min after stimulation with argon generated CAP for 180 s a maximum increase of p53 phosphorylation has been seen, returning to a baseline level within 24 h [29]. There seem to be differences in the regulation of keratinocytes and osteoblast-like cells, different application times or the CAP device used. Further experiments are necessary to investigate how plasma affects activation of transcription factors. A main effector of p53 is APAF-1 [30]. The downregulation of this apoptotic marker in osteoblast-like cells after CAP treatment underlines the protective effect of CAP. Similar CAP effects have also been described in PDL cells [17]. In contrast, the CAP treatment of dysplastic cells like leukaemia or melanoma led to an upregulation of p53 [23, 31]. The cellular response of the osteoblast-like cells we used in our study declare, that there seem to be different responses in different cells – maybe depending on intensity and duration of CAP application. In the studies mentioned above, the cells were treated for different periods: Arndt et al. has exposed cells to CAP for 120 s, Turrini et al. for 10 s, and Gümbel et al. for 60 and 120 s. All the authors used different CAP devices. Different cell types or different CAP devices might possibly induce different cellular responses regarding CAP treatment. Cold plasma could therefore be useful for various, even opposing medical purposes, such as wound healing on the one hand and tumour therapy strategies on the other. Further research is needed to establish an optimal therapeutic approach.

Additionally, we focused on the CAP-induced downregulation of CASP9 and CASP3. CASP3 plays an important role during the initiation of apoptosis [32]. The protein itself is processed and activated by CASP9, which is crucial for the apoptotic protease cascade [33]. Apoptosis is regulated by various signaling pathways. The MEK/ERK signaling pathway regulates the activation of apoptosis under circumstances of e.g. DNA damage [34]. A significant involvement of the MAP kinase signaling pathway in the effects of an indirect CAP treatment with argon generated plasma has already been shown by other authors: Schmidt et al. observed a CAP-dependent activation of MAP kinase signaling effectors, such as anti-apoptotic Hsp27, growth factors and cytokines [29]. Inhibition of the MAP kinase pathway by the U0126 inhibitor led to a reduction of the anti-apoptotic CAP effect. Additionally, we investigated the PI3 kinase signaling pathway. Other authors have described, that CAP effects were mediated by the PI3 kinase pathway as well [35, 36]: As described by Adhikari et al., CAP mediates PI3 kinase-mediated reduction of the mTOR gene, leading to a reduction in apoptosis inhibition in human melanoma cells [36]. Blocking PI3 kinase caused an inhibition of the CAP effect. As mentioned above, CAP appears to have different effects in different cells, possibly depending on the device, the used gas or a different application time. The fact that the CAP-induced downregulation of CASP9 can be counteracted by both inhibitors confirms the hypothesis that multiple pathways are responsible for the CAP effects. Further studies, possibly by blocking the corresponding signaling pathways simultaneously, are necessary to better understand the effects of CAP. With regard to the study of Schmidt et al., the differences in the significance of the signaling pathway could be due to the different cells and the different way of CAP generation [29]. Further studies are necessary to point this out. Our results concerning downregulation of CASP3 indicate positive effects of CAP treatment for osteoblast-like cells. Schmidt et al. observed similar results in keratinocytes after 20 s of CAP treatment. However, for longer treatments up to 180 s an induction of CASP3 was observed [29]. In contrast, an upregulation of CASP3 in LNCaP cells after 10 s of argon CAP application was observed by Weiss et al. [37, 38]. Nevertheless, the difference in CASP3 regulation after CAP treatment could be associated due to differences in cell types and different application times. Due to the fact that we have observed differences in the regulation of apoptotic genes in osteoblast-like cells compared to the cells described in the literature, a comprehensive study of anti-apoptotic genes in malignant cells would be interesting. Further studies have to clarify the parameters of CAP treatment and its effect on different cells.

It has also been considered that both inhibiting and stimulating signals contribute to the apoptotic cascade in a biologically balanced manner. Antiapoptotic genes, such as BCL2, Hsp72 or Hsp90 have been described to be upregulated in cells resistant to apoptosis [39]. For this reason, we focused on the anti-apoptotic gene BCL2 and one of its antagonists BAK1. We observed an upregulation of BCL2 gene expression, which was time-dependent after 30 resp. 60 s of CAP treatment at one day. Similar results were observed in leukaemia cells, with a further increase in BCL2 expression after 120 s of CAP exposure compared to 60 s [23]. Interestingly, Turrini et al. also show an increase in apoptotic cells by CAP using flow cytometry analysis and an increase in pro-apoptotic markers that was not detectable in our study. Once again, there is evidence that different cells can react differently to CAP. Further studies could help to illustrate the CAP mediated regulation of anti-apoptotic genes.

The effect of CAP on bacteria is well known, being both effective to reduce bacterial load on surfaces and tissues. [40, 41]. Clinical studies have shown a significant reduction of microbes in wounds and also a reduction in the time required for wound closure by argon CAP [42, 43]. Another aspect of chronic wounds is the persistence of inflammation and apoptotic processes. For single simulation of CAP effects on cells and tissues in an inflamed environment, representing chronic wounds, we studied the expression of IL-1β pre-treated osteoblast-like cells after CAP treatment. IL-1β has been shown to be increased in inflamed gingival tissues [44, 45]. In previously published studied the preincubation of IL-1β resulted in an upregulation of different markers, such as COX2, CCL2, IL6, IL8 or MMP1 [46–48]. Our study provides evidence that IL-1β-induced upregulation of apoptosis markers can be significantly attenuated by CAP application. This could indicate that the reduction of apoptotic activity of cells in chronic wounds could also be a reason for the surface reduction of chronic wounds after CAP treatment. Further clinical studies are necessary to clarify its effect on infected wounds. In addition, it must be taken into account that we only used GAPDH as a reference gene, which remained constant in our experiments and has also been described by other authors for the study of mRNA expression after CAP application [17, 27, 29, 49, 50]. Finally, it should also be kept in mind that we only analysed mRNA expression after 24 h. Additional investigations on longer time points should be the subject of further studies.

For evaluation of morphologic changes and viability of cells, phalloidin and DAPI staining were used. CAP-stimulated cells did not show apoptotic signs after 24 h as compared to the control, but had higher number of lamellipodia and filopodia. The cytoskeletal change of more filopodia and lamellipodia is characteristic of increased migration [51, 52]. Cell migration as a fundamental mechanism involved in wound repair requires higher cell deformation [53]. In a previous study we observed the effect of CAP on cell migration of osteoblast-like cells *in vitro*, shown by an XTT assay and a higher wound-fill-rate - therefore our results confirm previous findings [27]. Other cell types like fibroblasts, epithelial cells and keratinocytes have also been described to show higher levels of cell migration after CAP exposure [14, 15, 54]. A higher cell migration is required for wound healing processes, which has been shown to be accelerated by CAP [55]. Different morphologic effects of CAP regarding application times and intensity parameters have been described in the literature. In a study of Eisenhauer and coworkers a high intensity CAP treatment caused a decreased protein expression and less lamellipodia as compared to lower intensity [56]. Future studies are necessary to understand its regulatory effect on cell morphology of different cell types and the involved molecular mechanisms.

For a quantification of apoptotic cells flow cytometry analysis was performed. Annexin V is used to stain cells that expose phosphatidylserine at the exterior of the cell membrane at the time of early apoptosis [57, 58]. Propidium iodide binds to DNA after disruption of the cell membrane and therefore functions as a marker for dead cells [59]. The combination of these dyes allows us to distinguish early apoptotic cells from necrotic cells. We even investigated that proliferation, migration and viability were promoted by CAP, as previously published results have shown [27]. Regarding our results mentioned above, CAP appears to have a positive influence on individual apoptotic genes and proteins, displaying that CAP is not only non-apoptotic but has an apoptosis-inhibiting effect. The differences in apoptosis between the gene analyses and the FACS analysis could be due to the different sensitivity of the assays. It is possible that the results of gene expression would also be detectable at the FACS level after a certain time.

Altogether, CAP can be derived from different gases. In our experiments we used a plasma device which operates with ambient air. CAP can also be generated using inert gases like argon or helium [60–62]. The management of ambient air devices facilitates for clinical application, because it does not require any additional gas supply. However, the composition of ambient air-generated CAP is more dependent from external factors like pressure and temperature instead of a consistent inert gas. Further studies should clarify which gas would be beneficial for clinical application.

## Conclusions

Short CAP application on osteoblast-like cells resulted in a reduced apoptosis and downregulated expression of apoptotic markers in an inflammatory and apoptotic environment. Additionally, CAP treatment led to changes in cell morphology. Therefore, our data suggest that CAP may serve patients with chronic hard-tissue wounds.

## Data Availability

The datasets used and/or analysed during the current study are available from the corresponding author on reasonable request.

## Abbreviations

CAP: Cold atmospheric plasma
APAF: Apoptotic protease activating factor
CASP: Caspase
BAK: BCL2 Antagonist/Killer
BCL: B-Cell Lymphoma
TNF: Tumor necrosis factor
ROS: Reactive oxygen species
DMEM: Dulbecco’s modified essential medium
FBS: Fetal bovine serum
IL: Interleukin
STS: Staurosporine
GAPDH: Glyceraldehyde 3-phosphate dehydrogenase
ECL: Enhanced chemiluminescence
